# Tetracyclines function as dual-action light-activated antibiotics

**DOI:** 10.1371/journal.pone.0196485

**Published:** 2018-05-09

**Authors:** Ya He, Ying-Ying Huang, Liyan Xi, Jeffrey A. Gelfand, Michael R. Hamblin

**Affiliations:** 1 Department of Dermatology, Sun Yat-sen Memorial Hospital, Sun Yat-sen University, Guangzhou, Guangdong, China; 2 Wellman Center for Photomedicine, Massachusetts General Hospital, Boston, Massachusetts, United States of America; 3 Department of Dermatology, Harvard Medical School, Boston, Massachusetts, United States of America; 4 Dermatology Hospital of Southern Medical University, Guangzhou, Guangdong, China; 5 Department of Medicine, Massachusetts General Hospital, Boston, Massachusetts, United States of America; 6 Harvard-MIT Division of Health Sciences and Technology, Cambridge, Massachusetts, United States of America; Universidade de Aveiro, PORTUGAL

## Abstract

Antimicrobial photodynamic inactivation (aPDI) employs photosensitizing dyes activated by visible light to produce reactive oxygen species. aPDI is independent of the antibiotic resistance status of the target cells, and is thought unlikely to produce resistance itself. Among many PS that have been investigated, tetracyclines occupy a unique niche. They are potentially dual-action compounds that can both kill bacteria under illumination, and prevent bacterial regrowth by inhibiting ribosomes. Tetracycline antibiotics are regarded as bacteriostatic rather than bactericidal. Doxycycline (DOTC) is excited best by UVA light (365 nm) while demeclocycline (DMCT) can be efficiently activated by blue light (415 nm) as well as UVA. Both compounds were able to eradicate Gram-positive (methicillin-resistant *Staphylococcus aureus)* and Gram-negative *(Escherichia coli)* bacteria (>6 log(10) steps of killing) at concentrations (10–50μM) and fluences (10-20J/cm^2^). In contrast to methylene blue, MB plus red light, tetracyclines photoinactivated bacteria in rich growth medium. When ~3 logs of bacteria were killed with DMCT/DOTC+light and the surviving cells were added to growth medium, further bacterial killing was observed, while the same experiment with MB allowed complete regrowth. MIC studies were carried out either in the dark or exposed to 0.5mW/cm^2^ blue light. Up to three extra steps (8-fold) increased antibiotic activity was found with light compared to dark, with MRSA and tetracycline-resistant strains of *E*. *coli*. Tetracyclines can accumulate in bacterial ribosomes, where they could be photoactivated with blue/UVA light producing microbial killing via ROS generation.

## Introduction

The alarming rise in antibiotic resistance has led to widespread predictions of the “end of the antibiotic era” [1]. The O’Neill report [2] predicted that by 2050 (if nothing were done to stem the growth of multi-drug resistant bacteria) there would have been 300 million premature deaths that would have cost the world economy $100 trillion. Therefore new approaches are required such as antimicrobial photodynamic inactivation (aPDI) [3, 4]. Photodynamic therapy (PDT) was discovered over 100 years ago [5], but since then has been mainly developed as a cancer therapy [6]. PDT is based upon the scientific principle that many dyes absorb light and can then interact with ambient oxygen to produce hydroxyl radicals (HO•) or singlet oxygen (^1^O_2_). These reactive oxygen species (ROS) can kill microbial cells by causing oxidative damage to their constituent biomolecules.

A surprisingly large number of chemical structures have been reported to be able to act as antimicrobial PS [7]. However, one class of chemical compounds that has not (up to now) been much studied as antimicrobial PS, is that of tetracycline antibiotics. The first report that tetracyclines could act as antibacterial PS was published as long ago as 1987 by Martin [8]. Since then there have only been two more reports about carrying out aPDI with tetracyclines [9, 10]. Tetracyclines function by inhibiting bacterial ribosomal protein synthesis [11], and are generally considered to be bacteriostatic rather than bactericidal, although this has been questioned [12]. Tetracyclines are selective for prokaryotic cells because they do not efficiently inhibit eukaryotic ribosomes [13] and their uptake by mammalian cells is less efficient than bacterial cells [14].

There have been several studies into the mechanisms of skin phototoxicity of tetracyclines, as phototoxicity is one of the major tetracycline side-effects [15].

Therefore the goal of the present study was to revisit whether tetracyclines could mediate aPDI, and possibly be dual-action light-activated antibiotics.

## Materials and methods

### Chemicals

Tetracycline (TC), Demeclocycline (DMCT), Doxycycline (DOTC), Minocycline (MC), and Methylene blue (MB) were purchased from Sigma-Aldrich (St. Louis, MO). TCs stock solutions were prepared in distilled H_2_O (dH_2_O) at 10mM (around 5 mg/mL) concentration prior to use. Solutions were stored at 4 °C in the dark for a maximum of 1 week. Phosphate-buffered saline (PBS) for microbial cells suspension and serial dilutions, Brain-heart infusion broth (BHI), and M63 salt medium for bacterial growth were purchased from Fisher Scientific (Waltham, MA).

### Light sources

A 365-nm UVA light-emitting diode (LED) light source (Larson Electronics LLC, Kemp, TX), a 415-nm blue light LED light source (Omnilux Clear-U, Glen Ellen, CA) and an incandescent light source with a 660 ± 15 nm band-pass filter fiber-optic probe (LumaCare, Newport Beach, CA). For light intensity measurements, a model IL-1700 research radiometer-photometer (International Light, Inc., Newburyport, MA) was used for the UVA light and a model DMM 199 power meter with 201 standard head (Coherent, Santa Clara, CA) was used for blue light and red light. Both the blue light, the red light and UVA sources could deliver a light spot covering six wells of a 24-well plate at an irradiance of 12mW/cm^2^ (1 J/cm^2^ delivered in 1.4 min).

### Absorption spectra and photodegradation

Stock solutions of tetracyclines were diluted in dH_2_O to 50μM using an Evolution^™^ 300 UV-Vis Spectrophotometer (Thermo Scientific, Waltham, MA). To assess the photostability of the TCs, the aqueous solutions were irradiated for 70 min at 365 nm for 50 J/cm^2^.

### Bacterial isolates

*Escherichia coli* (UTI 89) (a kind gift from Patrick C Seed, Duke University Medical Center) and methicillin-resistant *Staphylococcus aureus* (USA300, ATCC BAA-1680). Two tetracycline-resistant *E coli* strains (5–66, 3–62, were kind gifts from David C. Hooper at Massachusetts General Hospital) used for MIC studies. Bacterial cells were cultured on BHI agar plates at 37°C. Cells were grown in BHI broth medium under shaking overnight to stationary phase. An aliquot of 1 mL from an overnight suspension was refreshed in BHI for 2h at 37°C before use. Cells were collected by centrifugation at 3,500 rpm for 5 min and suspended in PBS at a density of 10^8^ cells/mL (estimated by measuring the optical density at 600 nm; 0.6 = 10^8^ cells/mL).

### Bacterial photoinactivation

A microbial cell suspension (10^8^ cells/mL) was mixed with different concentrations (0, 0.1, 1, 5, 10, 50 μM) of TCs. After incubation in the dark at 37°C for 30 minutes, 200 μL of the suspension was transferred to 48-well plates. According to the absorption spectra results (shown in Results section), cells were illuminated at room temperature using blue light for DMCT and MC, and UVA light for DOTC and TC with 10 J/cm^2^. To further study the bactericidal activities of photosensitized DMCT and DOTC, 10μM DMCT or DOTC was irradiated with either BL or UVA at different light doses (0, 5, 15, 20, 25 J/cm^2^). After exposure to the calculated dose of irradiation, 10 μL aliquots were withdrawn, 10-fold serially diluted in PBS, and streaked on BHI agar plates according to the method of Jett et al [16]. CFU were counted after overnight incubation at 37°C.

To detect the binding of TCs to bacterial cells, the mixtures of DMCT or DOTC and cells were centrifuged at 4000 rpm for 5 mins and re-suspended with PBS. The original mixtures and washed suspensions were irradiated with 10 J/cm^2^ BL (for DMCT) or UVA (for DOTC).

Comparison of photoinactivation with DMCT, DOTC and MB were carried out in different media namely PBS, M63 salt medium (2% glycerol) and BHI. Cell suspensions (10^8^cells/ml) were mixed with DMCT/DOTC (10 μM) or MB (8 μM for *E coli*, 4 μM for MRSA) were divided into three and incubated in these three media for 30 min and irradiated with 10J/cm^2^ of BL, UVA, RL respectively.

Cell suspensions (10^8^ cells/mL) mixed with DMCT/DOTC (5 μM) or MB (8 μM for *E coli*, 4 μM for MRSA) was incubated in PBS for 30 min, irradiated with 10 J/cm^2^ BL, UVA, RL, and 10μL aliquots were withdrawn to count CFU. Then 500 μL aliquots of the remaining suspension were added to 500 μL of fresh M63/glycerol medium and incubated overnight at 37°C with shaking. 500 μL unirradiated cell suspension was also added into M63 medium as a growth control. 10μL aliquots of each incubated suspension were sampled to count CFU.

#### Antibiotic susceptibility tests in light or dark (MIC values)

Antimicrobial susceptibility was evaluated using the broth dilution method in accordance with The Clinical and Laboratory Standards Institute [CLSI] (2015) [17]. BHI medium was used. Four TCs were tested: DMCT or DOTC (2.5–0.005 μg/mL for *E coli* and MRSA; 64–0.125 μg/mL for TC-resistant strains), TC or MC (5–0.005 μg/mL for *E coli* and MRSA). Two identical 96-well plates for each agent were prepared. One was continuously exposed to 0.5 mW/cm^2^ blue light while the other was incubated in the dark at 37°C. After 16h irradiation or incubation, absorbance was measured with a microplate spectrophotometer (Spectra Max M5, Molecular Devices) at 610 nm.

### Statistics

Values are present as means and error bars are SD. Significance (p<0.05) was determined by a two-tailed unpaired t-test.

## Results

### Spectra and photostability

**Fig 1:**
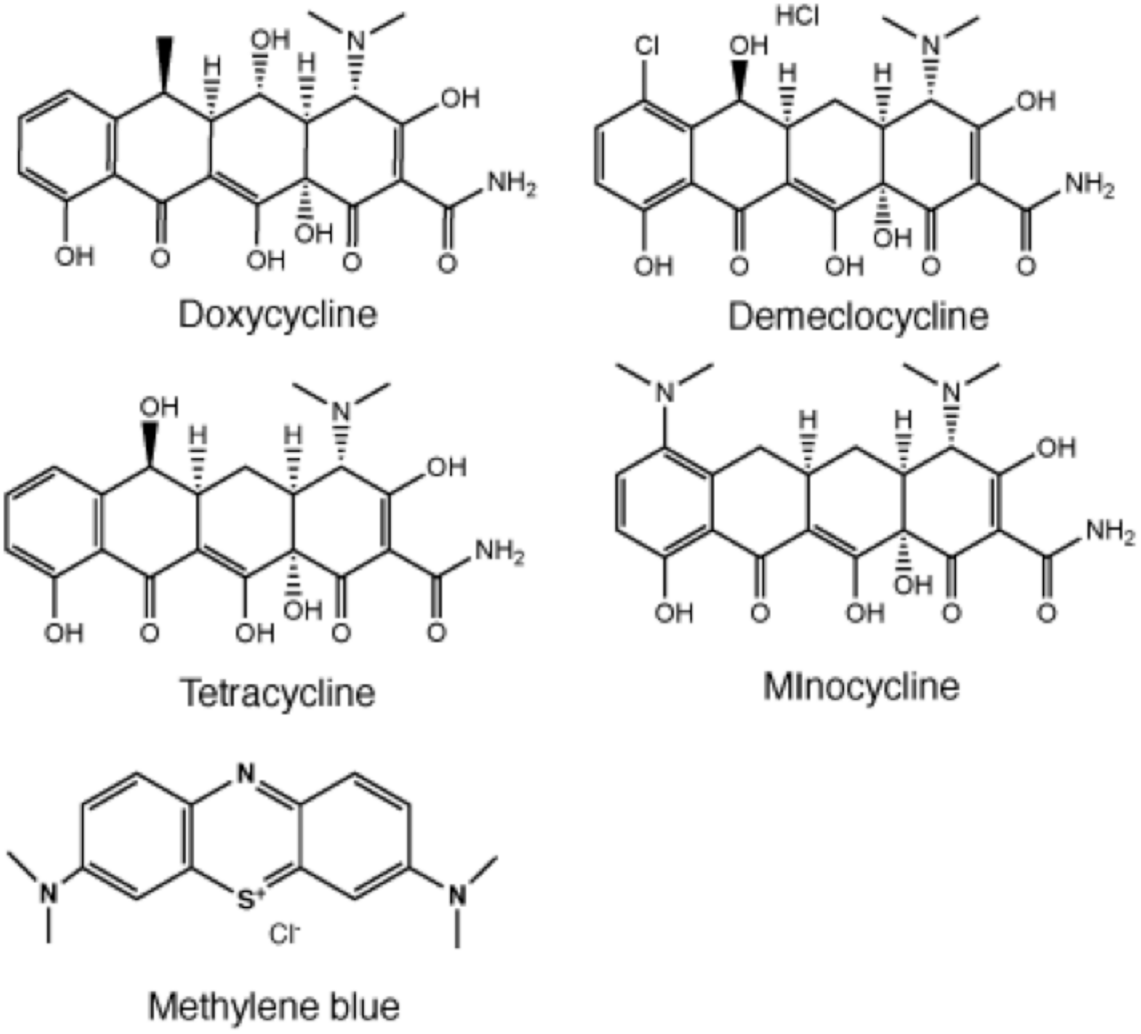
Chemical structures of tetracyclines together with methylene blue.

**Fig 2:**
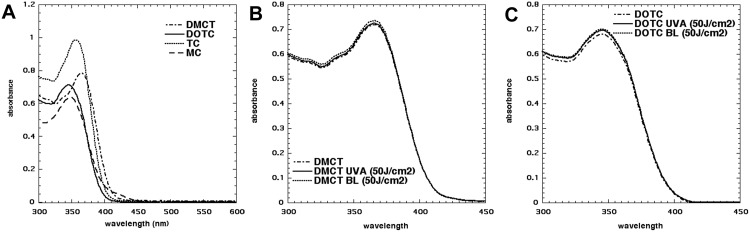
Absorption spectra and photostability. (A) Absorption spectra of DMCT, DOTC, TC and MC at 50 μM in PBS. (B) Absorption spectra of DMCT before and after irradiation with 50 J/cm^2^ of UVA or BL. (C) Absorption spectra of DOTC before and after irradiation with 50 J/cm^2^ of UVA or BL.

The chemical structures of the four tetracyclines are shown in Fig 1. To choose the optimum wavelength of light the absorption spectra of DMCT, DOTC, TC and MC were measured at 50μM. In order to assess the photostablitity of TCs, absorption spectra of DMCT and DOTC were measured before and after exposure to both BL (50 J/cm^2^) and UVA (50 J/cm^2^). The spectra before irradiation are shown in Fig 2A. The values of λmax and ε(max) are given in Table 1, together with the ε-values at 415 nm (BL). While all TCs are efficiently excited by UVA light, DMCT would also be able to be efficiently excited by BL. We did not detect any photobleaching after delivery of 50 J/cm^2^ of either wavelength (Fig 2B and 2C).

**Table 1:** UV-Vis absorption parameters.

	λ(max) nm	ε(max) L.mol^-1^ cm^-1^	ε(415) L.mol^-1^ cm^-1^
DMCT	366	15640	840
DOTC	346	14240	80
TC	357	19700	260
MC	349	12720	1120

### In vitro killing of *E coli* UTI89 and MRSA using DMCT or MC excited by blue light, and DOTC or TC excited by UVA light

**Fig 3:**
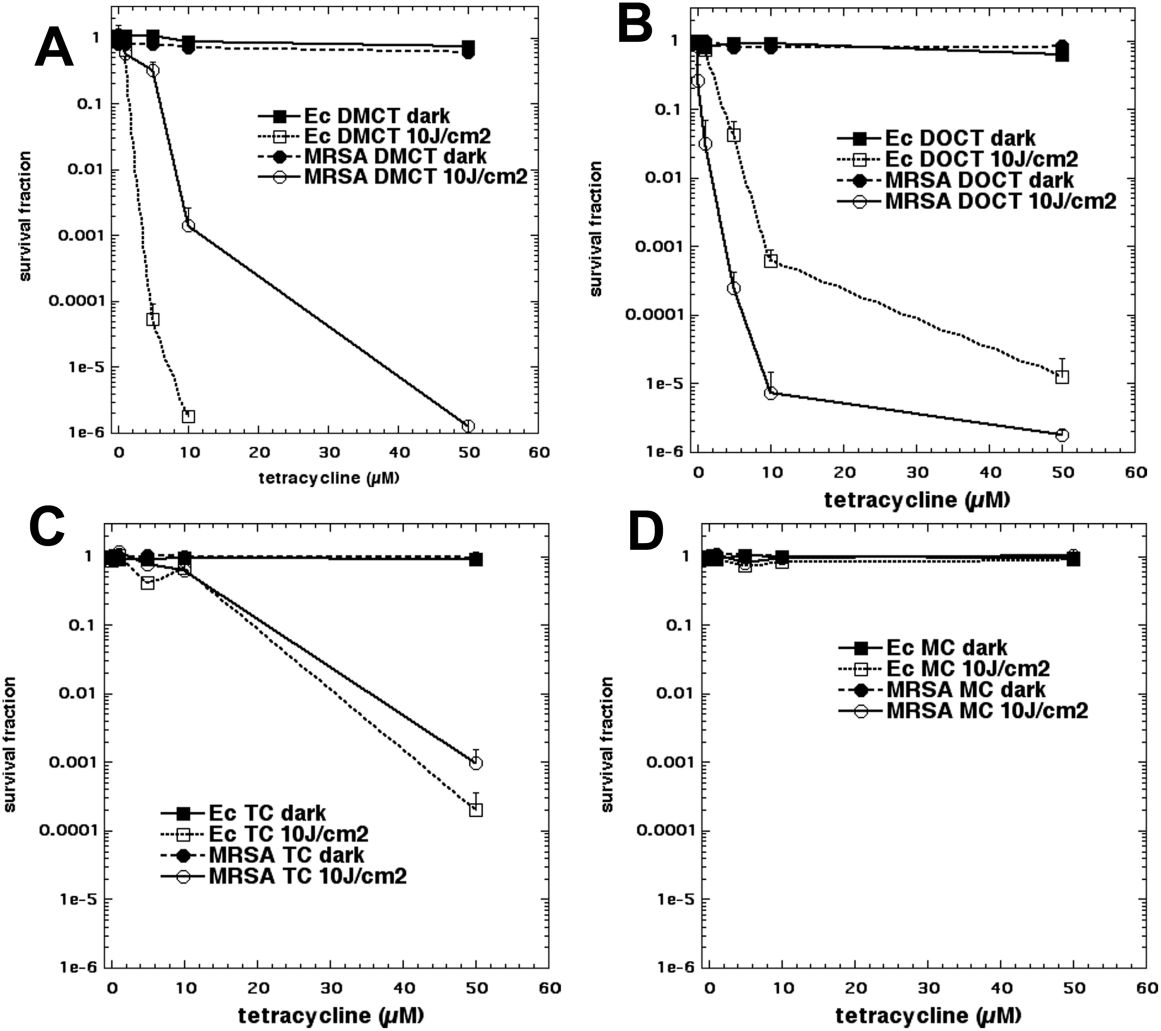
aPDI of two strains of bacteria by four tetracyclines. Bacterial cells were incubated for 30 min in PBS with stated concentration of tetracycline and exposed to 10 J/cm^2^ of the appropriate light. (A) DMCT and BL; (B) DOCT and UVA; (C) TC and UVA; (D) MC and BL.

None of the bacteria showed any significant phototoxicity (< 1 log killing) when irradiated with up to 20 J/cm^2^ of UVA light or blue light alone (data not shown). We initially tested four different tetracyclines. Two of them (DMCT and MC) were activated with blue light (415 nm), while the other two compounds (DOTC and TC) were activated by UVA light (365 nm). Fig 3 shows the killing curves obtained by varying the tetracycline concentration from 0.1–50 μM and exposing the cells *(E*. *coli* or MRSA) to fixed doses of the relevant wavelength (either BL or UVA; dark or 10 J/cm^2^). In Fig 3A *E*. *coli* was eradicated (6 logs of killing) by 10μM DMCT plus 10 J/cm^2^ BL, while MRSA required 50 μM. Fig 3B shows that DOTC plus UVA eradicated MRSA at 50 μM, while only about 4.5 logs of *E*. *coli* were killed. In Fig 3C the corresponding curves are shown for TC plus UVA, but both species were substantially less sensitive with only about 3–4 logs of killing being obtained. In Fig 3D MC was excited by blue light but absolutely no bacterial killing was seen, even at the highest doses of tetracycline. In view of these data, it was decided to conduct further studies on DMCT activated by BL, and with DOTC activated by UVA.

**Fig 4:**
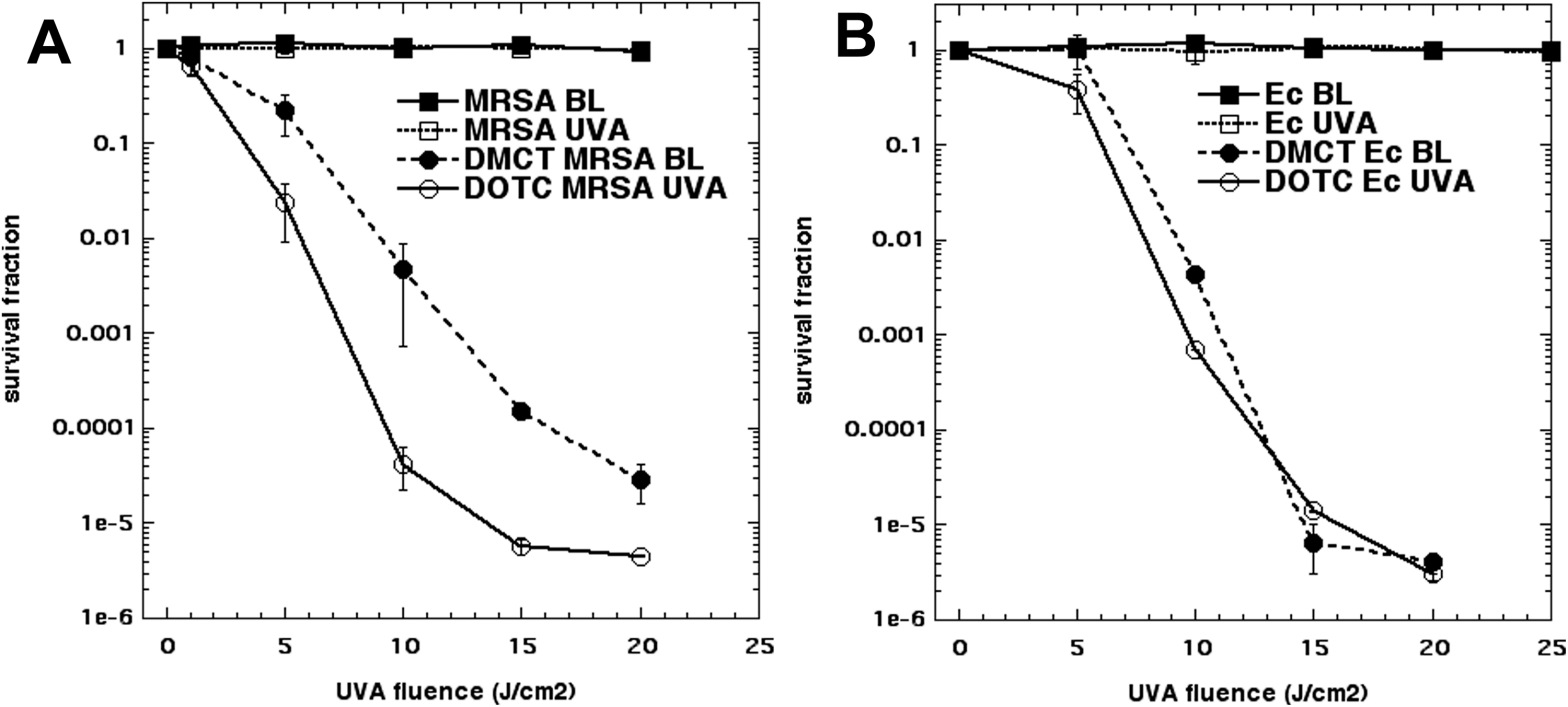
Light-dose dependent aPDI. Bacterial cells (*E*. *coli* UTI89 or MRSA) were incubated for 30 min in PBS with 10 μM tetracycline (DMCT or DOTC) and exposed to increasing fluences (J/cm^2^) of the appropriate light (UVA or BL) or to light alone controls. (A) MRSA; (B) *E*. *coli*.

Next we designed experiments to show that the degree of bacterial killing was dependent on the light dose delivered (J/cm^2^). We used 10 μM of either DMCT or DOTC and delivered increasing fluences of light. In Fig 4A it can be seen that *E coli* was substantially more sensitive (compared to MRSA) to DMCT excited by BL (in agreement with Fig 3A). In Fig 4B it can be seen that using DOTC and UVA, that MRSA was somewhat more sensitive than *E*. *coli* (in agreement with Fig 3B).

**Fig 5:**
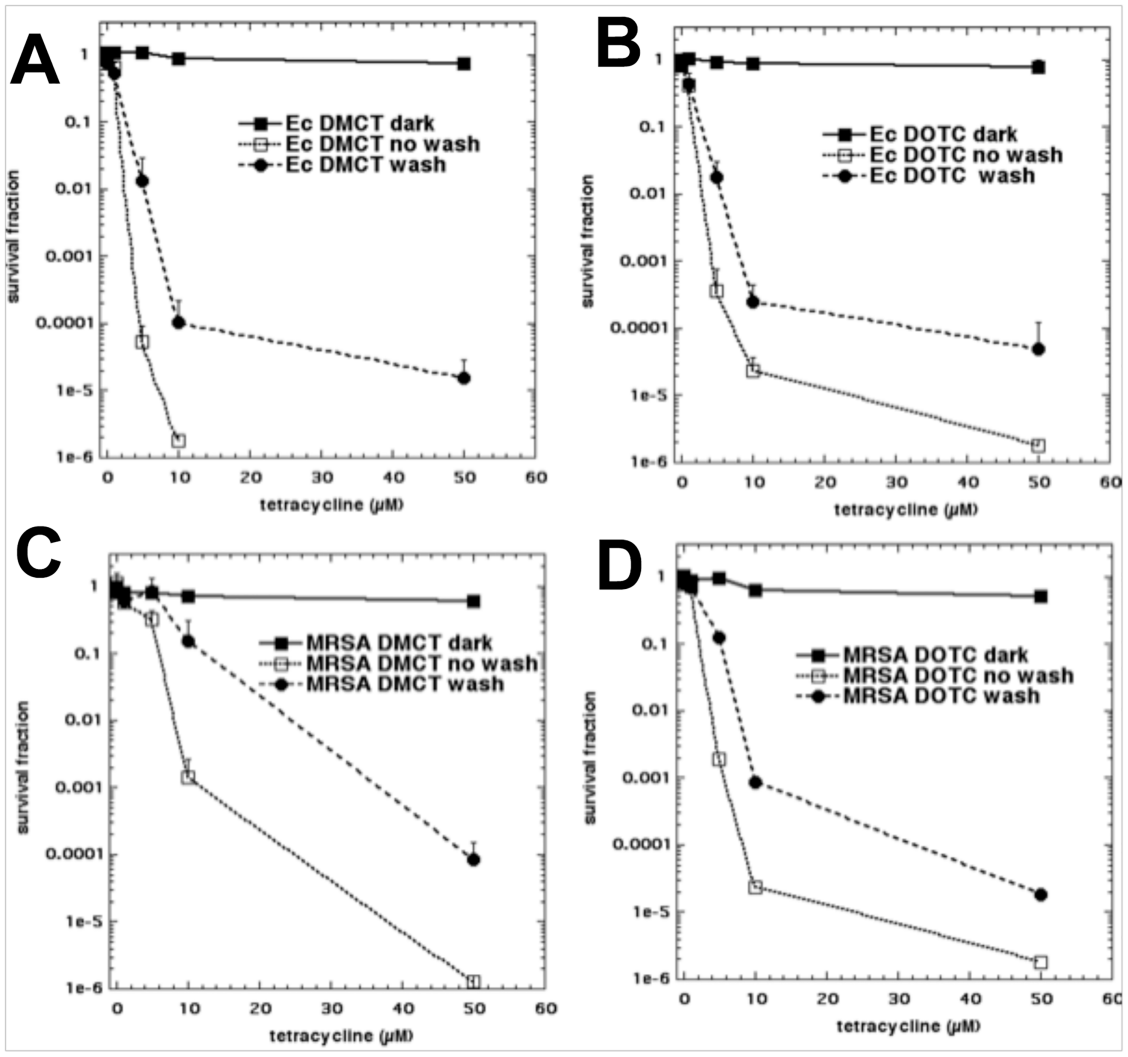
Effect of a wash-step on aPDI with tetracyclines. Bacterial cells were incubated for 30 min in PBS with stated concentration of tetracycline and either exposed (or not “dark”) to 10 J/cm2 of the appropriate light (“no wash”), or centrifuged and resuspended in fresh PBS and exposed to 10 J/cm2 (“wash”). (A) *E*. *coli* UTI89, DMCT and BL; (B) *E*. *coli* UTI89, DOCT and UVA; (C) MRSA, DMCT and BL; (D) MRSA, DOTC and UVA.

We next asked whether the tetracyclines could bind to the bacterial cells after a centrifugation and resuspension step (“wash”). The results are shown in Fig 5. In every case (Fig 5A–5D) there was still substantial bacterial killing remaining after a wash step. The killing after a wash was only slightly less (1–2 logs) than the killing found with the “no wash” protocol.

### Killing in PBS, M63 salt medium, BHI, compared with MB/RL

It has long been known that the vast majority of aPDI experiments are much more efficient if the incubation of the bacteria cells with the PS takes place in PBS as compared to bacterial growth medium. Although the reasons for this difference are not completely understood, one hypothesis is that many PS bind preferentially to proteins present in growth media (especially rich media such as BHI), and this binding prevents the PS from binding and penetrating the microbial cells. Another possibility is that the ROS produced during photoactivation, preferentially react with oxidizable groups present on these biological molecules, in other words the growth medium acts as a ROS quencher. However if tetracyclines penetrate to the bacterial ribosomes, the ROS they produce there, may be much less affected by proteins present in the surrounding medium compared with ROS produced by other PS that bind to the exterior of the bacterial cells.

**Fig 6:**
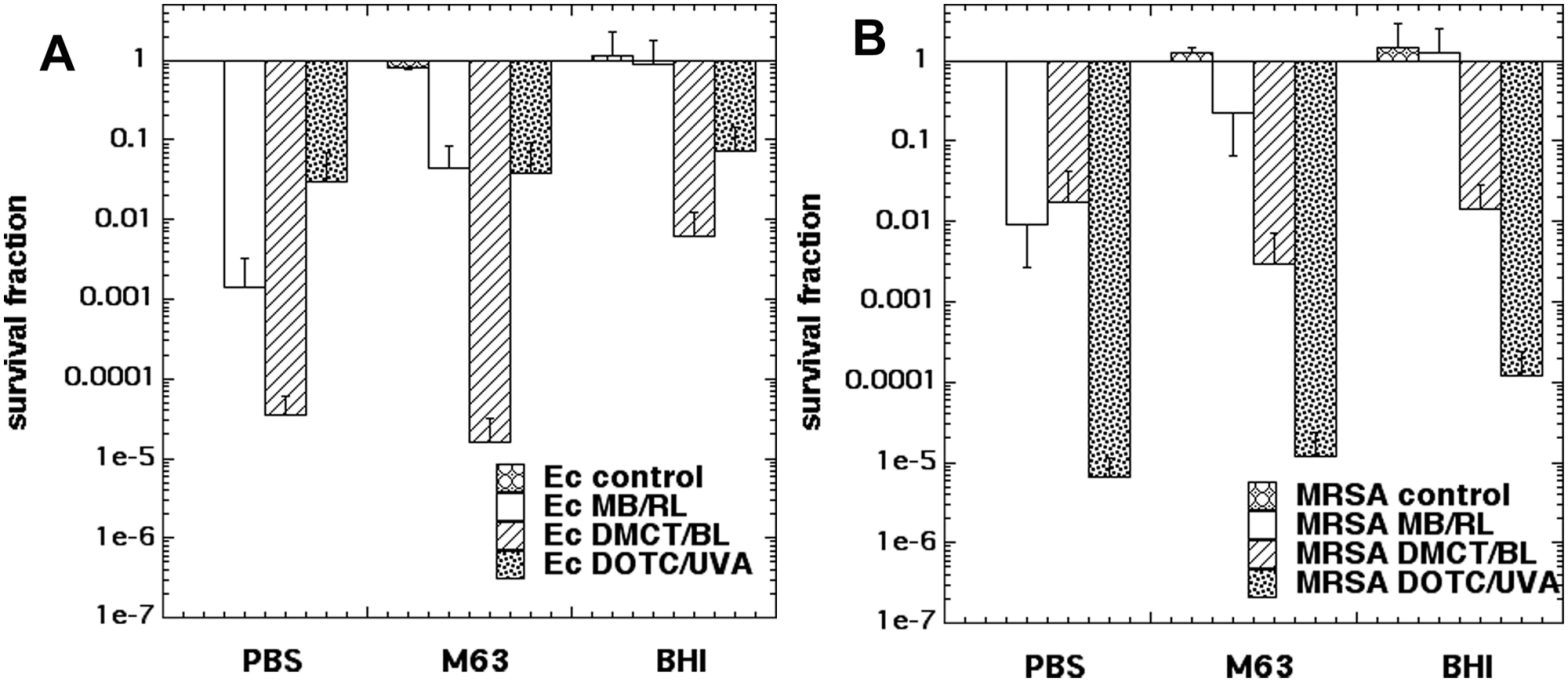
Effect of different incubation media on aPDI. Bacterial cells were incubated for 30 minutes in one of three different media with DMCT or DOTC at 10 μM, or with MB at 8 μM (for *E*. *coli* UTI89) or 4 μM (for MRSA), and exposed to 10 J/cm2 (taking 14 min) of the appropriate light (360 nm for DOTC; 415 nm for DMCT; 660 nm for MB). (A) *E*. *coli* UTI89; (B) MRSA.

We therefore compared aPDI with DMCT/BL and DOTC/UVA with a traditional cationic PS, MB and red light. We incubated the cells with the PS in three different media namely PBS, M63 salt medium and BHI. Fig 6A shows that when *E*. coli was treated with aPDI using the chosen parameters in PBS, MB/RL gave 3 logs of killing; DMCT/BL gave 4.5 logs, and DOTC/UVA gave 1.5 logs. When the experiment was repeated in the salt medium with glucose (M63) the killing with MB/RL was reduced to only just over 1 log, while the killing obtained with DMCT/BL and DOTC/UVA was practically unchanged. When the medium was switched to the protein-rich BHI, the killing produced by MB/RL was completely abolished. The killing produced by DMCT/BL was reduced but still significant in BHI, while the killing caused by DOTC/UVA was hardly affected. Fig 6B shows analogous experiments carried out with MRSA. Here MB/RL gave 2 logs of killing in PBS, which was reduced to less than 1 log in M63 and again completely abolished in BHI. By contrast there was no difference for both DMCT/BL and DOTC/UVA in M63 medium, and only a slight reduction for both combinations in BHI.

### Partial killing in PBS and regrowth in BHI compared with MB

**Fig 7:**
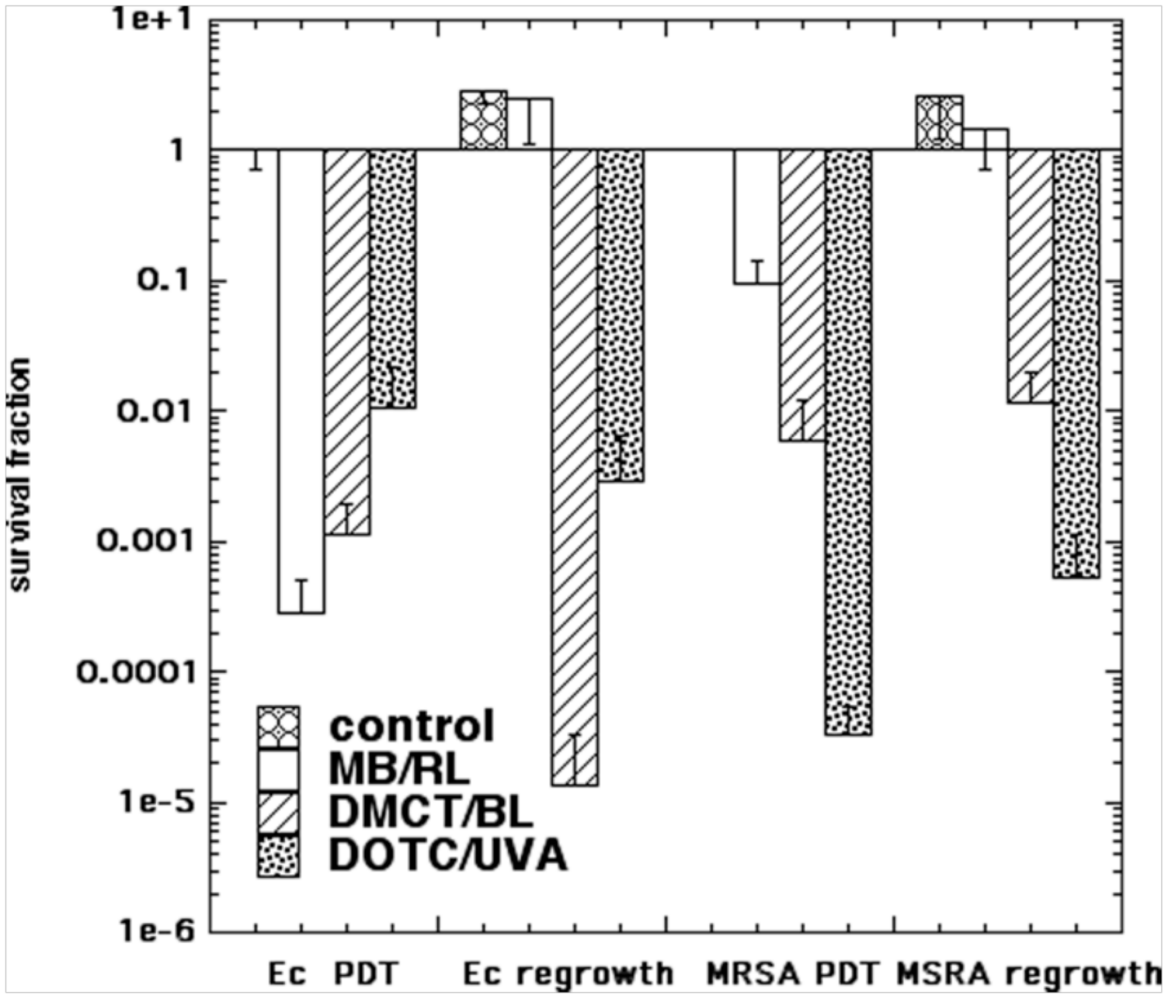
Effect of regrowth in medium after aPDI in PBS. Bacterial cells (*E*. *coli* (Ec) or MRSA were incubated in PBS with DMCT or DOTC at 5 μM, or with MB at 8 μM (for *E*. *coli* UTI89) or 4 μM (for MRSA), and exposed to 10 J/cm^2^ of the appropriate light (BL for DMCT; UVA for DOTC; RL for MB). The samples were assayed for CFU and then added to fresh growth medium and incubated overnight and the CFU were determined again.

One of the attractions of using an antibiotic as an antimicrobial PS, is that when the light is switched off (after rapidly killing several logs of bacteria), the antibiotic is expected to keep on exerting its designed function and inhibiting the bacteria from growing (provided the antibiotic has not been totally destroyed). We chose aPDI parameters to kill several logs (but not eradication) of *E*. *coli* and MRSA using the combinations (MB/RL; DMCT/BL; DOTC/UVA) in PBS. After the light, the CFUs remaining were sampled, and then 500 μL remaining suspension was added to 500 μL of fresh M63 medium and incubated overnight at 37°C with shaking. The results are shown in Fig 7. For *E*. *coli* the highest number of logs of killing (3.5) was obtained for MB/RL, but after regrowth in BHI, the CFUs had completely recovered to a stationary value (4 logs of regrowth). By contrast, with DMCT/BL *E*. *coli* had undergone about 3 logs of killing immediately after aPDI, and this number of CFUs had actually been further reduced (another 2 logs of killing) to give 5 logs total after regrowth. The same situation was found with *E*. *coli* and DOTC/UVA where a value of 2 logs of killing immediately after aPDI, underwent a slight further reduction by 0.5 log. With MRSA MB/RL killed cells underwent complete regrowth, while cells that had been killed with aPDT mediated by DMCT/BL or DOTC/UVA hardly showed any regrowth.

### MICs measured in light and dark

**Fig 8:**
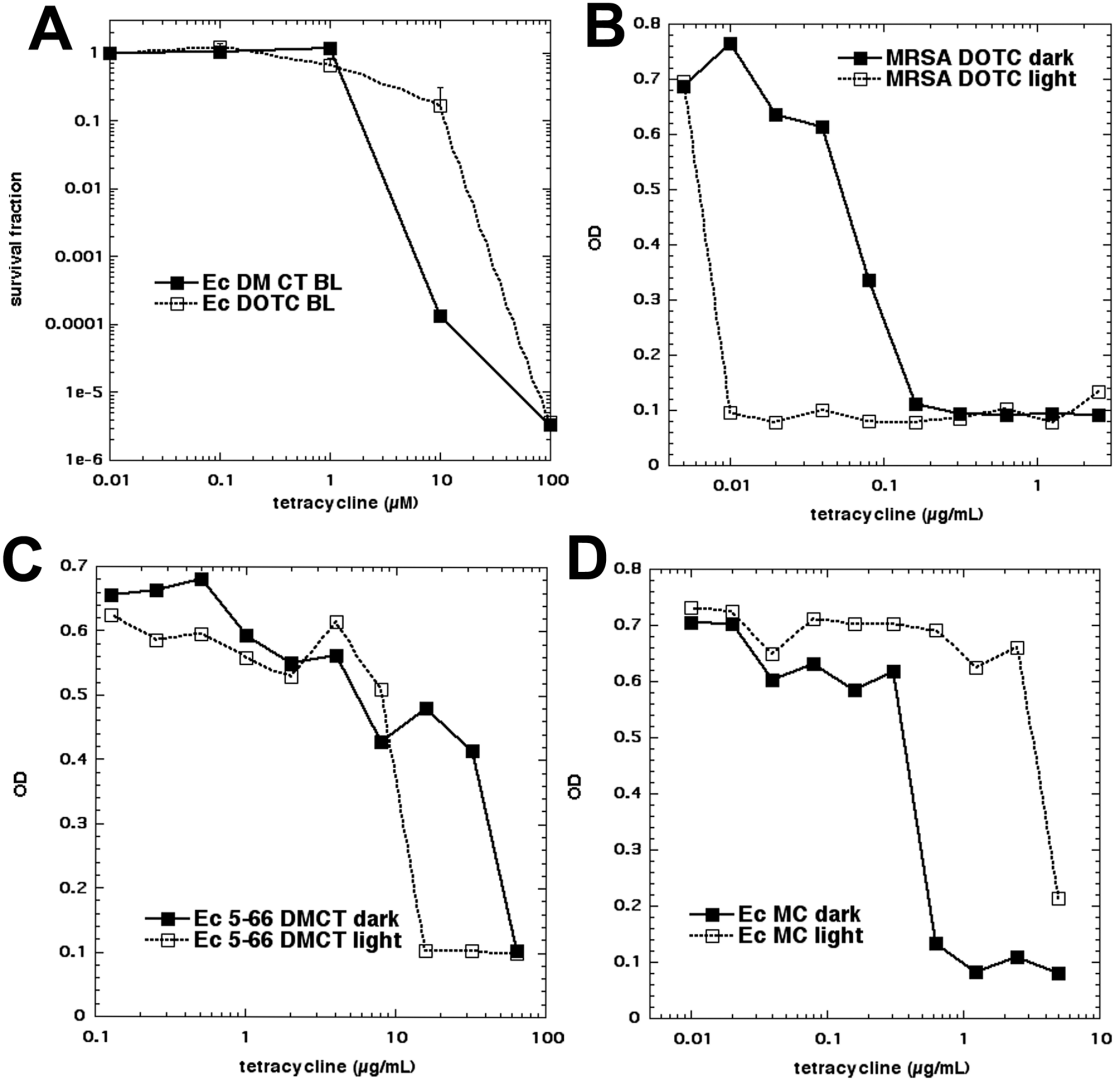
MIC determination in light and dark. (A) Initially we tested whether both DMCT and DOTC could be activated with BL. Next a standard broth microdilution assay (16 h) was conducted with incubation either in dark or exposed to 0.5 J/cm2 BL. Three examples are given. (B) MRSA with DOTC; (C) *E*. *coli* (5–66 resistant) with DMCT; (D) *E*. *coli* (UTI 89) with MC.

Since the tetracyclines allowed aPDI in bacterial growth medium, we asked whether the traditional MIC values would be affected by the presence of light for the entire 16-hour growth period, instead of just the few minutes required to carry out aPDI. Because we only were able to use BL for this prolonged irradiation protocol (the UVA lamp would not be robust enough) it was necessary to determine how effective BL was in activating DOTC (which we had hitherto always activated with UV light). Therefore Fig 8A shows *E*. *coli* killed by activation of both DMCT and DOTC by BL. As expected the DOTC activation by BL was less efficient than UVA light, but nevertheless eradication was achieved with 100 μM and 10 J/cm^2^. We then proceeded to carry out standard broth-microdilution MIC determinations with *E*. *coli* and MRSA and DMCT, DOTC, TC and MC. Fig 8B–8D shows three examples, and Table 2 summarizes the results together with statistics. The tetracycline-susceptible *E*. *coli* strain (UTI89) did not show any difference between the MIC values of DMCT (0.16 μg/mL), DOTC (0.625 μg/mL), or TC (1.25 μg/mL) when cultured in the dark or when irradiated with 0.5 mW/cm^2^ of 405 nm light for 16 h (29 J/cm^2^). For MC we found a remarkable difference (in the wrong direction) in that the MIC in the dark (0.32 μg/mL) was substantially lower than that in the light (>5 μg/mL). This result requires further explanation, especially because we could find no evidence that MC (or indeed any tetracycline) was substantially photobleached or photodegraded by this fluence of light. For MRSA the results were more promising. The MICs for DMCT dropped from 0.08 μg/mL (dark) to 0.02 μg/mL (light); for DOTC from 0.08 μg/mL (dark) to 0.01 μg/mL (light); and for TC from 0.16 μg/mL (dark) to 0.08 μg/mL (light). The MIC for MC was unchanged at 0.04 μg/mL. We then asked whether it was possible that application of light could reverse tetracycline in a drug-resistant strain. The *E*. *coli* strain 5–66 showed a MIC value for DMCT of 64 μg/mL in the dark, which was reduced to 16 μg/mL in the light, and for DOTC a MIC value of 16 μg/mL in the dark was reduced to 4 μg/mL in the light. A similar result was found in the second resistant stain *E*. *coli* 3–62, where a MIC value of DMCT of 64 μg/mL in the dark was reduced to 16 μg/mL in the light, and for DOTC from 16 μg/mL in the dark to 8 μg/mL in the light. If DOTC is taken as the reference (1.0 photon absorption efficiency) and the molar concentration and absorption coefficient at 415 nm are considered, DMCT shows approx., a 33-fold photon absorption factor, TC shows a 44-fold photon absorption factor, and MC a 91-fold photon absorption factor, which makes DOTC, comparatively, much more photoactive against MRSA than DMCT and TC. Moreover this order DMCT (33) > TC (44) > MC (91) correlates with the decreasing observed values of the singlet oxygen quantum yields for these compounds (0.08 > 0.05 and nearly 0, respectively) see the Discussion section.

**Table 2:** Light and dark MIC values for *E*. *coli*, MRSA, 2 tetracycline resistant *E*. *coli* strains using 4 tetracyclines.

Microorganism	Tetracycline	Dark MIC (μg/mL) ^a^	Light MIC (μg/mL)	P value
*E coli* UTI	DMCT	0.16–0.32	0.16–032	n.s.
	DOTC	0.625	0.625	n.s.
	TC	1.25	1.25	n.s.
	MC	0.32–0.625	>5	<0.0001
MRSA	DMCT	0.08	0.02–0.04	0.0001
	DOTC	0.08–0.16	0.01–0.02	0.0003
	TC	0.16–0.32	0.08–0.16	0.018
	MC	0.04	0.04	n.s.
*E*. *coli* 5–66	DMCT	64	16–32	0.0002
	DOTC	16	4–8	0.0134
*E*. *coli* 3–62	DMCT	64	16–32	0.0004
	DOTC	16–32	8	0.0134

P values determined by 2-tailed unpaired t-test.

^a^ as is customary values are given in μg/mL. In order to compare with concentrations given in μM in Fig 3 it should be noted that 1μg/mL is equivalent to 2.25 μM for DOTC and 2 μM for DMCT.

It should be emphasized that this experiment was different from the previous experiments, which aimed to kill several logs of bacteria starting from an initial population of 10(8) cells/mL. Here the aim was to prevent bacterial growth starting from a low initial density of 10(4) cells/mL, and therefore an overnight growth period was employed.

## Discussion

The present study has shown that tetracyclines can act as dual-action light-activated antibiotics. This beneficial feature may be able to overcome one of the most troubling drawbacks of aPDT when it is used as a therapy for localized infections. This consists of the fact that once the light is switched off, the photochemical production of highly bactericidal (but very short-lived) ROS ceases, and there is nothing (except possibly the host immune system) to stop the bacteria growing back. Although all tetracyclines (except MC) were activated by UVA light (365 nm), DMCT was also efficiently activated by blue light (415 nm). The absorption spectrum of DMCT, as well as its ability to act as a photosensitizer when administered to mice by the oral route or injected either intraperitoneally or intradermally was reported by Stratigos and Magnus as long ago as 1967 [18].

Tetracyclines were some of the first clinically used antibiotics after they were discovered in 1945 by Duggar [19]. The first compound was aureomycin isolated from *Streptomyces aureofaciens*, and later renamed chlortetracycline. Second-generation semisynthetic analogs and more recent third-generation compounds have been synthesized with increased potency and improved pharmacokinetic and chemical properties [20].

We did not find any photobleaching of the tetracyclines after 50 J/cm^2^ of either UVA or BL. Several other workers have reported on the photostability (or lack thereof) of different TCs. Hasan et al [21] reported that DMCT, DOTC, and TC were bleached by doses of UVA as low as 4 J/cm^2^, while MC was fairly photostable. Goldman et al studied [22] photoincorporation of tetracyclines into *E*. *coli* ribosomes, to identify which proteins acted as the binding sites (photoaffinity labeling [23]). They found three separate processes: photoincorporation of native tetracycline, photoincorporation of tetracycline photoproduct, and light-independent incorporation of tetracycline photoproduct. Niu et al reported that TC [24] was considerably more photostable than found by Hasan [21]. It is possible that differences in the precise wavelength distribution within the emission spectrum of the light source could explain some of these differences.

Martin et al [8] studied the ability of four different tetracyclines (DMCT, DOCT, TC, and oxytetracycline) to kill *E*. *coli* in glucose minimal medium, when excited by UVA light (130 μW/cm^2^ for 70 min). The order of effectiveness was DOCT > DMCT > TC > oxytetracycline, while although the concentrations were higher than the present work (up to 160 μM), the light fluences were lower. Martin et al found that the photochemical mechanism of action was Type 1 (using pre-induction of intracellular superoxide dismutase and catalase to inhibit killing), or addition of hydroxyl-radical scavengers [8].

Hasan and Khan [21] reported that the mechanism of action of tetracyclines in causing skin photoxicity was Type 2, involving photosensitized production of “singlet delta dioxygen”. They calculated the singlet oxygen quantum yields to be DMCT = 0.08; TC = 0.05; and MC = 0.00. The lack of activity of MC found by these workers was in agreement with our finding that MC was essentially without any activity as an antibacterial PS. It is at present not entirely clear which structural features make DMCT, DOTC and TC photodynamically active, while MC is not.

There are substantial differences between tetracyclines and more conventional antimicrobial photosensitizers, such as MB [25]. MB is a cationic phenothiazinium dye that binds to anionic residues on the outside of bacterial cells such as lipotechoic acids (Gram-positives) or lipopolysaccharides (LPS) (Gram-negatives). In Gram-negative cell walls MB can displace divalent cations (Ca^2+^ and Mg^2+^) leading to destabilization of the LPS in the so-called “self-promoted uptake pathway” [26]. Tetracyclines however, are taken up into Gram-negative cells via the OmpF and OmpC porin channels, in the form of complexes with positively charged cations [11].

This active uptake and targeting of tetracyclines to internal organelles within the bacterial cells makes these compounds fudamentally different from the majority of antimicrobial PS. The efficiency of tetracyclines is not much affected by incubation in protein-rich BHI growth media, while for MB the aPDI activity is totally abolished. With DMCT/BL, the Gram-negative *E*. *coli* showed much more killing than MRSA. Other PS have been shown to kill Gram-positive bacteria more easily, because the permeability barrier of Gram-negative bacteria inhibits uptake [27]. Thirdly, the “dual function” nature of tetracyclines allowed us to compare the effects of killing some logs of cells with tetracycline aPDI, and then to add the whole partly killed suspension to growth medium. In the case of *E*. *coli* it appeared that the PDT treated bacteria were even more sensitive to the bactericidal action of tetracyclines, as 2 more logs were killed by DMCT and < 1 log with DOTC (Fig 6). This could be of great clinical importance, as the main problem encountered in treating localized infections by PDT, is the likelihood of regrowth occurring in the infected tissue following an apparently successful initial killing of several logs of bacterial cells. It is likely that the tetracycline remaining in the infected area (after the light was switched off) would inhibit this regrowth. Another very interesting possibility is that conventional oral administration of tetracycline pills may allow a sufficient concentration of PS in the infected area to carry out aPDI. Since a tetracycline concentration of 1 μg/mL (pharmacologically active) is ~2 μM which can kill several logs in the case of a susceptible combination (e.g. *E*. *coli*, DMCT/BL). The only drawback to possible clinical application of aPDT using tetracyclines, is the activation wavelength (UVA or BL). Nevertheless we have previously published successful PDT treatment using UVA-mediated excitation of functionalized fullerenes in a mouse abrasion wound infected with bioluminescent *Acinetobacter baumannii)* [28].

The MIC values improved when the bacterial cultures containing tetracyclines were illuminated, but only in the case of resistant *E*. *coli* or MRSA, but not tetracycline-susceptible *E*. *coli* UTI. Further study is required to explain these differences. It should be noted that the overnight incubation time with BL (16h) was intended only to prove the point that the antibiotic activity could be potentiated by light, and not as a possible clinical treatment protocol.

It should also be noted that there have been several publications suggesting that aPDI can be advantageously combined with a variety of antibiotics and that these combinations may even be synergistic [29–32].

In summary, we have demonstrated substantial reductions in the MIC values of tetracyclines by activating them with light. Our findings suggest that localized infections by highly resistant organisms could be rendered treatable by the use of aPDT mediated by the antibiotics themselves.
